# Development and Psychometric Evaluation of the Military Cadets’ Mental Health Risk Scale (MCMHRS) in Chinese Military Academy Cadets

**DOI:** 10.3390/healthcare14172730

**Published:** 2026-08-27

**Authors:** Feifei Wang, Jia Guo, Guoyu Yang

**Affiliations:** 1 Department of Developmental Psychology of Armyman, School of Psychology, Army Medical University, Chongqing 400038, China; 2 School of Psychology, Army Medical University, Chongqing 400038, China; 3 School of Culture and Tourism, Chongqing Business Vocational College, Chongqing 401331, China

**Keywords:** scale development, psychometric evaluation, mental health assessment, confirmatory factor analysis, risk–resource framework

## Abstract

**Background**: Military academy cadets face training, study, and adaptational pressures that elevate the risk for mental health problems and functional impairment. Existing tools are either generic symptom checklists or narrow resilience scales; none integrates environmental risk, individual vulnerability, and protective resources within a unified hierarchical model relevant to this population. **Methods**: We developed and psychometrically evaluated the Military Cadets’ Mental Health Risk Scale (MCMHRS). After item generation and two-round Delphi review (*n* = 15), a 64-item pool was administered to 610 Chinese cadets (mean age 19.45 ± 1.42 years; 82.6% male), randomly split into calibration (*n* = 244) and validation (*n* = 366) subsamples for exploratory and confirmatory factor analyses and reliability/validity testing against the PHQ-9, GAD-7, and WHO-5. **Results**: The final 53-item scale comprised 13 first-order factors nested within three second-order dimensions, with excellent fit (RMSEA = 0.043, CFI = 0.978), strong internal consistency (*α* = 0.968), and coherent correlations with PHQ-9 (*r* = 0.70), GAD-7 (*r* = 0.69), and WHO-5 (*r* = −0.58). **Conclusions**: The MCMHRS shows initial evidence of being a psychometrically sound, culturally relevant, multilevel assessment tool that may support early identification and tiered intervention in cadet mental healthcare pending further longitudinal validation.

## 1. Introduction

Cadets in military academies are in a developmental and vocational situation that combines the demands of emerging adulthood with the intensity of a highly regimented training environment. Along with the academic and developmental pressures that all college students face, cadets are also expected to adapt to militarized daily schedules, limited personal autonomy, rigorous physical training, and explicit behavioral codes governing conduct both on and off duty, all of which may tax the psychological resources required to maintain resilience in military populations [1,2]. These stresses may elevate cadets’ risk of psychological distress, suicidal ideation, and attrition [3,4]. Although international studies have reported high levels of mental health risk in military cadet populations, systematic epidemiological data specifically describing the prevalence and patterning of mental health problems among Chinese military academy cadets are still scarce, partly because of the lack of population-specific assessment instruments.

Contemporary models of mental health risk increasingly integrate three theoretical strands: the diathesis–stress interaction between vulnerability and environmental stress [5], the developmental resilience perspective [6], and the resource-conservation framework that views protective assets as partially independent buffers against stress [7]. Recent work conceptualizes mental health risk as a dynamic balance between exposure, vulnerability, and resources [8]—a perspective particularly congruent with the cadet experience, in which acute and chronic stresses co-occur with institutional, interpersonal, and intrapersonal resources for adjustment. However, the translation of this risk–resource concept into multidimensional assessment instruments for cadet populations remains lacking.

Existing instruments for cadet mental health fall into three types, each with notable limitations. Symptom checklists such as the SCL-90 capture distress but were designed for general psychiatric assessment rather than the specific stress ecology of military training. Resilience-oriented tools such as the Connor–Davidson Resilience Scale, although validated in Chinese military samples [9], measure protective traits only. Stress-focused inventories quantify environmental demands but omit individual vulnerability and buffering resources. Reliance on multiple instruments to cover these complementary domains increases respondent burden and limits integrated risk profiling.

Reliable, culturally appropriate measurement is a prerequisite for evidence-based mental healthcare. Beyond symptom counting, contemporary measurement frameworks increasingly emphasize upstream risk and resource configurations that predict functional outcomes such as social adjustment, training engagement, and occupational continuity [10]. For cadets, whose future occupational functioning is heavily contingent on early adaptation, an instrument capturing the joint configuration of stressors, vulnerabilities, and resources—rather than downstream symptomatology alone—would directly support early identification, tiered intervention, and longitudinal monitoring in routine institutional practice.

Against this background, the present study developed and psychometrically evaluated the Military Cadets’ Mental Health Risk Scale (MCMHRS) in Chinese military academies. Specifically, our aims were: (a) to generate and refine an item pool grounded in the diathesis–stress–resource framework through literature review, focus groups, and a two-round Delphi procedure; (b) to examine the factor structure using exploratory and confirmatory factor analyses on independent calibration and validation subsamples; and (c) to evaluate convergent, discriminant, and criterion-related validity against established measures of depression, anxiety, and well-being. An interpretable hierarchical structure was expected, although the exact number and arrangement of factors were treated as empirical issues to be resolved by the data.

## 2. Materials and Methods

### 2.1. Study Design

The instrument development process involved four phases: (a) literature review, focus groups, and theoretical mapping to generate an item pool; (b) content validation using a two-round Delphi expert procedure; (c) exploratory factor analysis (EFA) on a calibration subsample; and (d) confirmatory factor analysis (CFA) and reliability/validity evaluation on an independent validation subsample. To prevent sample reuse bias, the participant pool was randomly split into two independent subsamples prior to any analyses, in accordance with psychometric instrument development recommendations [10]. The study was approved by the Medical Ethics Committee of the Army Medical University (Approval No. 2025-3-02; 16 May 2025) in accordance with the Declaration of Helsinki.

### 2.2. Participants and Procedures

Data were collected at a Chinese military academy from 15 September to 15 October 2025. Through stratified convenience sampling across three academic years, 643 cadets were recruited; inclusion criteria were enrolled cadet status, age ≥ 18 years, and voluntary informed consent. All 643 questionnaires were returned, and three pre-specified exclusion criteria (completion time < 3 min, failed attention checks, or patterned responding) yielded a final analytic sample of 610 cadets (94.87%). The 3 min threshold corresponds to fewer than 2 s per item across the 91-item survey (64 MCMHRS items, PHQ-9, GAD-7, WHO-5, and six demographic items), below the empirical lower bound for meaningful response and thus indicative of careless responding [11].

The questionnaire was administered online through Wenjuanxing (www.wjx.cn) in dedicated computer rooms; each session took approximately 25 min. To safeguard voluntary participation within the academy setting, no unit commanders or instructors were present during data collection, and research assistants remained outside the room while cadets completed the survey. Before accessing items, participants reviewed a standardized informed consent screen detailing the study purpose, anonymity, and explicitly stating that refusal or withdrawal would carry no academic, disciplinary, or career consequences; electronic consent was provided by clicking “I agree to participate.” IP-based duplicate-submission control was activated, and no personally identifying information was collected.

The final sample (*N* = 610; mean age 19.45 ± 1.42 years, range 18–25; 82.6% male) is described in Table 1. The total sample was randomly partitioned into a calibration subsample (*n* = 244; 40.0%) for EFA and a validation subsample (*n* = 366; 60.0%) for CFA using the SPSS random number generator (seed = 20250901). The 40:60 split allocated a larger proportion to the validation subsample to ensure adequate power for CFA [12]. Demographic equivalence between subsamples was confirmed by an independent-samples *t*-test for age (*t* = 0.268, *df* = 608, *p* = 0.788) and chi-square tests for categorical variables (all *p* > 0.05).

### 2.3. Item Pool Development

The original item pool was generated through four successive processes. We first conducted a systematic literature analysis to identify constructs and indicators that might be relevant for mental health risk in military academy cadets. Constructs and indicators were found using the diathesis–stress–resource framework [5,6,7]. Second, focus groups with 18 cadets and 8 instructors identified population-specific stressors, vulnerabilities, and resources, guided by a semi-structured interview protocol; two researchers with backgrounds in military psychology independently coded the transcripts using an inductive–deductive approach, with discrepancies resolved by discussion. Third, an initial pool of 78 potential items was written, divided into three a priori theoretical dimensions: environmental stressors (D1), individual vulnerabilities (D2), and protective factors (D3). Items were drafted in simplified Chinese with attention to readability and avoidance of double-barreled phrasing.

Content validity was established using a two-round Delphi procedure [10] with 15 experts (mean experience 16.4 years; specialties: military psychology *n* = 6, clinical psychology *n* = 5, psychometrics *n* = 4). At each round, experts appraised the relevance of the items on a 4-point scale (1 = not relevant, 4 = extremely relevant) and offered qualitative input on wording, comprehensibility, cultural appropriateness, redundancy, and conceptual coverage. The item-level content validity index (I-CVI) was calculated as the proportion of experts rating an item 3 or 4 [13], with a retention threshold of I-CVI ≥ 0.78 [14]. Items below threshold were revised or removed, and new items were added when conceptual gaps were detected. Across the two Delphi rounds, 17 items were removed for low I-CVI or substantive redundancy, 6 were substantially reworded, and 3 new items were added. I-CVI values ranged from 0.80 to 1.00, the scale-level content validity index (S-CVI/Ave) was 0.93, and Kendall’s coefficient of concordance was *W* = 0.42 (*p* < 0.001), indicating acceptable expert agreement. The final 64-item pool was scored on a 5-point Likert scale (1 = strongly disagree, 5 = strongly agree).

### 2.4. Measures

#### 2.4.1. Military Cadets’ Mental Health Risk Scale (MCMHRS)

The 64-item MCMHRS pool was administered as outlined in Section 2.3. MCMHRS items are analyzed in their original direction throughout the study: higher D1 and D2 scores indicate greater environmental stressors and individual vulnerability, and higher D3 scores indicate greater protective resources. When a single composite total is reported, D3 items are reverse-coded before summation so that a higher total score consistently reflects greater overall mental health risk.

#### 2.4.2. Patient Health Questionnaire-9 (PHQ-9)

Depressive symptoms over the preceding two weeks were assessed with the Chinese version of the PHQ-9 [15,16]. To match the 1–4 Likert format used in the accompanying institutional questionnaires, items were administered on a 1–4 scale and linearly transformed to the standard 0–3 scoring system (total 0–27) prior to analysis. Higher scores indicate greater symptom severity. Cronbach’s *α* in the present sample was 0.878.

#### 2.4.3. Generalized Anxiety Disorder-7 Scale (GAD-7)

Anxiety symptoms over the preceding two weeks were assessed with the Chinese version of the GAD-7 [17,18], administered and rescaled in the same manner as the PHQ-9 (total 0–21). Higher scores indicate greater anxiety severity. Cronbach’s *α* in the present sample was 0.910.

#### 2.4.4. WHO-5 Well-Being Scale (WHO-5)

Subjective well-being over the last two weeks was measured using the Chinese version of WHO-5 [19]. The 5 items were rated on a 6-point scale (1 = all of the time, 6 = at no time), reverse-coded so that higher scores indicate greater well-being, and rescaled to the standard 0–25 range (0–100 in clinical reporting). Descriptive statistics in Section 3.6.3 are reported on the 0–100 clinical-reporting scale. Cronbach’s *α* in the present sample was 0.952.

### 2.5. Statistical Analysis

All analyses were performed using IBM SPSS Statistics 26.0 and Mplus 8.3 [20]. We used the criterion of *α* = 0.05 (two-tailed) for statistical significance. A thorough explanation of analytic techniques, decision criteria, and supporting references is included in Supplementary Table S1.

#### 2.5.1. Item Analysis

Item analysis on the calibration subsample (*n* = 244) applied two complementary criteria: critical ratio (top vs. bottom 27%; retained if CR ≥ 3.0, *p* < 0.001) and corrected item-total correlation (retained if *r* ≥ 0.30) [21]. Final retention integrated empirical and theoretical considerations.

#### 2.5.2. Exploratory Factor Analysis (EFA)

EFA was performed independently for each of the three a priori dimensions on the calibration subsample (*n* = 244). Sampling adequacy was confirmed by KMO (≥0.70) and Bartlett’s test (*p* < 0.001). Principal axis factoring with Promax oblique rotation (kappa = 4) was used [22]. Factor retention combined eigenvalues ≥ 1.0, a scree plot, parallel analysis [23], and theoretical interpretability. Items were retained if the primary loading was ≥0.40, the cross-loading difference ≥ 0.20, and communality ≥ 0.20 [24].

#### 2.5.3. Confirmatory Factor Analysis (CFA)

CFA was conducted on the validation subsample (*n* = 366) using Mplus 8.3 with the WLSMV estimator [25]. Three competing models—unidimensional (M0), 13-factor correlated (M1), and hierarchical second-order (M2)—were compared. Model fit was evaluated using χ^2^/df, RMSEA, CFI, TLI, and SRMR against conventional thresholds [26] (see Supplementary Table S1). Nested-model comparisons used ΔCFI < 0.01, ΔRMSEA < 0.015, ΔSRMR < 0.030 [27]. Final model selection balanced empirical fit with theoretical parsimony.

The 53 items were treated as ordered categorical indicators, with delta parameterization. Because mean- and variance-adjusted χ^2^ statistics are not distributed as χ^2^ for difference testing, the nested comparison of M1 and M2 was conducted using the DIFFTEST procedure implemented in Mplus [28], which yields a correctly scaled difference statistic for WLSMV. As a further index specific to hierarchical models, the target coefficient [29]—the ratio of the first-order to the higher-order χ^2^—was computed to quantify the proportion of first-order factor covariation explained by the second-order structure. Because likelihood-based information criteria are undefined under WLSMV, all three models were additionally re-estimated with maximum likelihood with robust standard errors (MLR), treating the five-point items as continuous, and Akaike’s information criterion (AIC), the Bayesian information criterion (BIC), and the sample-size-adjusted BIC (aBIC) are reported for these solutions, with lower values indicating a more favorable balance of fit and parsimony.

#### 2.5.4. Reliability

On the validation subsample, reliability was assessed using Cronbach’s *α* [21], Spearman–Brown split-half coefficients, composite reliability [30], and average variance extracted [31], with the latter two computed from CFA standardized loadings. Conventional thresholds were applied (*α* ≥ 0.70; split-half ≥ 0.70; CR ≥ 0.70; AVE ≥ 0.50; Supplementary Table S1).

#### 2.5.5. Validity

Convergent validity was evaluated via CFA standardized loadings, CR, and AVE [32]. Discriminant validity was assessed by the Fornell–Larcker criterion [31] and, at the second-order level, complementary HTMT ratios [33]. Criterion-related validity was examined by Pearson correlations between MCMHRS scores and the PHQ-9, GAD-7, and WHO-5 in the validation subsample (*n* = 366). D1 and D2 were expected to correlate positively with PHQ-9/GAD-7 and negatively with WHO-5, while D3 (original direction) was expected to show the reverse pattern and negative correlations with D1/D2.

## 3. Results

### 3.1. Participant Characteristics

Demographic characteristics of the total sample (*N* = 610) are presented in Table 1. The calibration (*n* = 244) and validation (*n* = 366) subsamples were demographically equivalent across all variables (all *p* > 0.05; Table 2), supporting the cross-validation design.

### 3.2. Item Analysis

Of the 64 initial items administered to the calibration subsample (*n* = 244), 63 met both retention criteria (all critical-ratio values > 3.0, *p* < 0.001; corrected item-total *r* ≥ 0.30). One item (C7) was excluded (corrected item-total *r* = 0.270; Cronbach’s *α* if deleted rose from 0.943 to 0.949), leaving 63 items for EFA. Full item-analysis results for all 64 initial items are reported in Supplementary Table S2.

### 3.3. Exploratory Factor Analysis

Sampling adequacy was excellent for all three dimensions (KMO = 0.894 for D1, 0.921 for D2, 0.928 for D3; Bartlett’s tests all *p* < 0.001).

Across the three dimensions, EFA yielded a stable 13-factor solution with 54 retained items. For D1 (26 items entered), six factors emerged (F1 military training pressure, F2 academic competition pressure, F3 military cultural adaptation, F4 interpersonal pressure, F5 career prospect pressure, F6 family-of-origin pressure), explaining 67.26% of the variance; A4, A16, and A23 were removed for cross-loading, leaving 23 items. For D2 (19 items entered), the a priori four-factor structure did not replicate; instead, three factors emerged (F7 emotional instability, F8 negative cognitive pattern, F9 behavioral impulsivity), with “maladaptive coping” items loading mainly on F8 (Section 4.2). Six items (B9, B10, B17–B20) were removed, leaving 13 items and 50.91% of the variance; B6 was subsequently removed following the CFA sensitivity analysis (Section 3.4), yielding 12 items. For D3 (18 items after C7 removal), four factors emerged (F10 psychological resilience, F11 social support, F12 professional identity, F13 positive coping), explaining 70.53% of the variance. Full sampling-adequacy and factor-retention details, together with the Promax-rotated pattern matrices and communalities for each dimension, are reported in Supplementary Tables S5–S8.

After EFA and CFA-based sensitivity analysis, the scale was shortened from the original 64 items to 53 items distributed across 13 first-order factors. A stage-by-stage summary of item reduction is provided in Table 3.

### 3.4. Confirmatory Factor Analysis (CFA)

Three competing CFA models were compared (Table 4): a single-factor baseline (M0), a 13-factor correlated model (M1), and the hypothesized second-order three-dimension model (M2).

The first-order 13-factor correlated model (M1) and the second-order three-dimension model (M2) both demonstrated excellent fit. Because M2 constrains the 91 parameters describing the factor-level covariance structure in M1 (13 factor variances and 78 factor covariances) to be reproduced by 29 parameters, it is strictly nested within M1 and imposes 62 restrictions, corresponding exactly to Δ*df* = 1309 − 1247 = 62 and to the difference in free parameters (339 − 277 = 62). The DIFFTEST comparison indicated that these restrictions produced a statistically significant deterioration in exact fit, Δχ2(62) = 236.490, *p* < 0.001. The χ^2^ difference test is, however, a test of exact fit and is highly sensitive to trivial misspecification at this sample size and model size; all approximate fit criteria indicated that the deterioration was negligible in practical terms (ΔCFI = 0.005, ΔRMSEA = 0.004, ΔSRMR = 0.013, all below conventional thresholds for a meaningful difference [27]). The target coefficient was T = 0.885, indicating that the three second-order dimensions accounted for 88.5% of the covariation among the 13 first-order factors.

**Figure 1:**
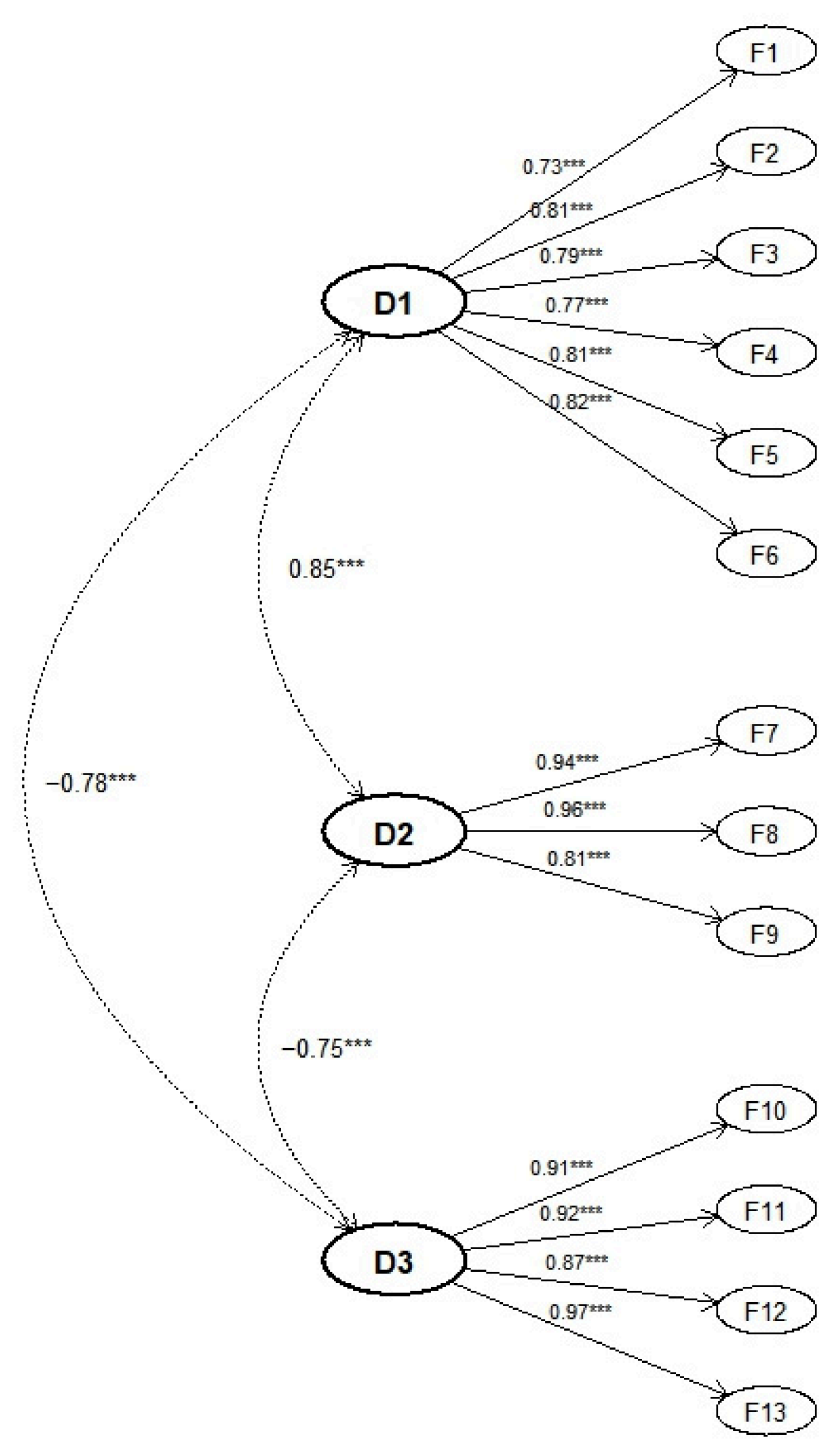
Path diagram of the second-order CFA model (M2; *n* = 366). *Note*. Solid arrows represent standardized second-order loadings; dashed double-headed arrows represent interdimension correlations. All paths are statistically significant at *p* < 0.001 (***).

Information criteria from the MLR re-estimation (Table 4, Panel B) were unequivocal in rejecting the unidimensional model, M0 exceeding both alternatives by more than 4800 units on every index. For the comparison of the two well-fitting models, the criteria diverged in the manner expected when a substantially more parsimonious model reproduces the data almost as well as its less constrained counterpart. BIC, whose complexity penalty increases with sample size, favored the second-order model M2 (37,011.553) over the first-order correlated model M1 (37,131.484) by ΔBIC = 119.9, a substantial difference favoring the more parsimonious specification; AIC (ΔAIC = 122.0) and aBIC (ΔaBIC = 76.8), whose penalties are weaker, favored M1. Weighing the negligible differences in approximate fit, the BIC advantage, the high target coefficient, the reduction of 62 free parameters, and the a priori diathesis–stress–resource framework, M2 was selected as the final model. A path diagram of the final second-order model is presented in Figure 1.

A sensitivity analysis compared the initial 54-item second-order specification with two alternatives: a 53-item variant with B6 removed and a 51-item variant with the entire F9 factor removed. Because this re-analysis was conducted in the same validation subsample (*n* = 366) used for the primary CFA, the resulting final specification should be regarded as provisional pending confirmation in an independent cadet sample. All three variants yielded excellent overall fit (54-item: RMSEA = 0.043, CFI = 0.977, SRMR = 0.058; 53-item: RMSEA = 0.043, CFI = 0.978, SRMR = 0.057; 51-item: RMSEA = 0.045, CFI = 0.978, SRMR = 0.057). Although overall fit was similarly good, the 53-item variant produced substantial improvements in F9 psychometrics (*α*: 0.596 → 0.788; CR: 0.776 → 0.876; AVE: 0.572 → 0.783; |*r*| with external criteria: 0.206–0.379 → 0.297–0.489) with high, balanced loadings (B7 β = 0.788; B8 β = 0.971), whereas the 51-item variant eliminated a theoretically meaningful construct without corresponding empirical gains. Accordingly, the 53-item second-order model was adopted as the final M2 specification, and M0 and M1 in Table 4 were re-estimated on the same 53-item indicator set to permit strictly like-for-like comparison.

All 53 first-order loadings in the final M2 model were statistically significant (*p* < 0.001), with standardized loadings ranging from 0.740 to 0.973. F9 in the final M2 model is indicated by B7 (β = 0.788) and B8 (β = 0.971); item B6, which had shown a notably low loading (β = 0.263) in an initial 54-item specification, was removed based on the sensitivity analysis described in Section 3.4. The 13 first-order factors were loaded on their respective second-order dimensions, with all loadings being statistically significant (*p* < 0.001; F7 = 0.936, F8 = 0.958, F9 = 0.812 on D2, and analogous loadings on D1 and D3). The three second-order dimensions were moderately to highly inter-correlated at the latent level (D1 ↔ D2 *r* = 0.850, D1 ↔ D3 *r* = −0.780, D2 ↔ D3 *r* = −0.746 after sign adjustment for D3, all *p* < 0.001), and HTMT ratios supported discriminant validity (see Section 3.6.2). No Heywood cases were observed. Full standardized loadings for all 53 items and the second-order loadings of the 13 first-order factors are presented in Supplementary Tables S3 and S4, respectively.

### 3.5. Reliability

The reliability indices at three levels are summarized in Table 5. Cronbach’s *α* ranged from 0.729 (F4) to 0.968 (full scale), with all 13 first-order factors ≥ 0.729 and all three second-order dimensions ≥ 0.926. Split-half, CR, and AVE values likewise met conventional thresholds (all CR ≥ 0.70; all AVE ≥ 0.50). Following the sensitivity analysis (Section 3.4), F9 achieved acceptable reliability (*α* = 0.788) with two indicators (B7, B8).

### 3.6. Validity

#### 3.6.1. Convergent Validity

All 13 first-order factors and three second-order dimensions met conventional thresholds for convergent validity (CR ≥ 0.70; AVE ≥ 0.50; Table 5).

#### 3.6.2. Discriminant Validity

Discriminant validity was evaluated using two complementary criteria. At the first-order level, the square root of AVE (√AVE) for each of the 13 factors exceeded the absolute value of its correlations with all other factors. At the second-order level (Table 6), the √AVE values for D2 (0.904) and D3 (0.918) each exceeded the absolute latent interdimension correlations, while √AVE for D1 (0.789) was marginally exceeded by the D1 ↔ D2 latent correlation (0.850) and only slightly above the absolute D1 ↔ D3 correlation (|−0.780|). As a complementary index, HTMT ratios [33] were computed for all three dimension pairs, and all fell well below the conservative 0.85 threshold (D1–D2 = 0.735; D1–D3 = 0.724; D2–D3 = 0.567), providing converging evidence for the empirical distinguishability of the three dimensions.

#### 3.6.3. Criterion-Related Validity

Pearson correlations between the MCMHRS and the three external criterion measures (PHQ-9, GAD-7, and WHO-5) in the validation subsample (*n* = 366) are presented in Table 7. All relationships were statistically significant (*p* < 0.001) and in line with theoretical predictions. D1 environmental stressors and D2 individual vulnerabilities correlated positively with PHQ-9 (*r* = 0.615 and *r* = 0.672, respectively) and GAD-7 (*r* = 0.605 and *r* = 0.679) and negatively with WHO-5 (*r* = −0.497 and *r* = −0.524). D3 protective factors exhibited the opposite pattern, correlating negatively with PHQ-9 (*r* = −0.583) and GAD-7 (*r* = −0.553) and positively with WHO-5 (*r* = 0.514). The full-scale total score correlated strongly with PHQ-9 (*r* = 0.700), GAD-7 (*r* = 0.685), and WHO-5 (*r* = −0.576). Of the 13 first-order factors, F7 emotional instability, F8 negative cognitive pattern, and F13 positive coping showed the strongest absolute correlations with the criterion measures (|*r|* = 0.496–0.664). F9 behavioral impulsivity, refined to two items after CFA-based sensitivity analysis, showed moderate associations (|*r*| = 0.297–0.489), consistent with its improved psychometric performance following B6 removal. Descriptive statistics for the criterion measures in the total sample (*N* = 610) were as follows (values in the validation subsample were essentially identical, given the random split): PHQ-9 *M* = 2.91 (*SD* = 3.42, range 0–25), GAD-7 *M* = 2.18 (*SD* = 3.10, range 0–21), and WHO-5 *M* = 77.41 (*SD* = 19.90, range 0–100), indicating adequate variability and no meaningful restriction of range.

## 4. Discussion

The present study developed and psychometrically evaluated the Military Cadets’ Mental Health Risk Scale (MCMHRS) in 610 Chinese military academy cadets, using independent calibration (*n* = 244) and validation (*n* = 366) subsamples. The final 53-item instrument comprises 13 first-order factors nested within three second-order dimensions—environmental stressors (D1), individual vulnerabilities (D2), and protective factors (D3)—and demonstrated excellent confirmatory fit, strong internal consistency, and theoretically coherent convergent, discriminant, and criterion-related validity. The following sections interpret these findings in light of the working hypotheses and prior literature, situate the instrument within the broader landscape of cadet mental health assessment, and consider its theoretical contributions, practical applications, limitations, and directions for future research.

### 4.1. The Hierarchical Risk–Resource Structure

Consistent with the open hypothesis stated in the Introduction, the results supported a hierarchical structure comprising 13 first-order factors nested within three second-order dimensions. This structure operationalizes, in a single instrument, the diathesis–stress, developmental resilience, and resource-conservation strands introduced earlier [5,6,7], aligning with contemporary conceptualizations of mental health risk as a dynamic equilibrium among exposure, susceptibility, and resources [8].

The latent correlations at the second-order level (D1 ↔ D2 = 0.850, D1 ↔ D3 = −0.780, D2 ↔ D3 = −0.746) are consistent with protective resources operating as a counterweight to—rather than the simple inverse of—risk exposure. The high D1–D2 latent correlation reflects the structural coupling between environmental stress exposure and individual vulnerability predicted by the diathesis–stress framework [5], while HTMT ratios below 0.85 confirm empirical distinguishability across all three dimensions (see Section 3.6.2 and Section 4.4).

M2 substantially outperformed the unidimensional alternative (M0) on every criterion and differed only marginally from the 13-factor correlated model (M1) in approximate fit (ΔCFI = 0.005, ΔRMSEA = 0.004, ΔSRMR = 0.013). It should nonetheless be noted that the formal χ^2^ difference test indicated a statistically significant loss of exact fit for M2 relative to M1, Δχ^2^(62) = 236.490, *p* < 0.001, and that AIC and aBIC favored M1 while BIC favored M2. This pattern is unsurprising rather than anomalous: the second-order model requires the 91 free elements describing the first-order factor covariance structure in M1 to be reproduced by 29 parameters, and with *n* = 366 the difference test has considerable power to detect even negligible departures, while AIC and BIC differ precisely in how heavily they penalize the 62 additional parameters that M1 expends. The selection of M2 therefore rests not on statistical equivalence with M1 but on the joint consideration of practically negligible differences in approximate fit, a decisive BIC advantage, a target coefficient of 0.885, substantially greater parsimony, and—most importantly—the theoretical interpretability of the three-dimension risk–resource structure, which is what confers the instrument’s intended multilevel scoring logic. Users whose purpose requires maximally precise reproduction of inter-factor relations may legitimately prefer the first-order solution.

### 4.2. Empirical Refinement of the Vulnerability Dimension

A major empirical revision was made to D2, where the initially proposed four-factor structure, which included a discrete “maladaptive coping” feature, failed to replicate. Instead, three coherent factors emerged: emotional instability (F7), negative cognitive pattern (F8), and behavioral impulsivity (F9), with items originally written to capture maladaptive coping loading largely on the negative cognition factor. This reorganization is consistent with transdiagnostic models that suggest that problems of emotion regulation, maladaptive cognition, and behavioral dyscontrol are interrelated rather than completely distinct processes [34,35]. It also suggests that, in cadets, what is usually called “coping” is mostly a cognitive-appraisal process rather than a distinct behavioral repertoire. The pattern is consistent with evidence from similar military samples that suggests emotion regulation in highly structured contexts is often characterized by clustering around cognitive reappraisal and rumination, with overt behavioral avoidance constrained by institutional norms [2,4].

This finding also illustrates the value of allowing a theoretically guided but empirically open EFA stage prior to confirmatory testing. Had the original four-factor solution been imposed through confirmatory means alone, both model fit and substantive interpretability would likely have suffered. The preserved three-factor structure is more parsimonious, fits the data well at the confirmatory stage, and is conceptually cohesive with the diathesis–stress paradigm.

### 4.3. Reliability and the Sensitivity Analysis for F9

Internal consistency was excellent at the total-scale (*α* = 0.968) and second-order dimension levels (*α* = 0.926–0.957). Extracted composite reliability and average variance further supported the reliability and convergent validity of the hierarchical structure [30,31]. All 13 first-order factors achieved *α* ≥ 0.729; F4 (interpersonal pressure, *α* = 0.729) and F6 (family-of-origin pressure, *α* = 0.743) reached the lower acceptable range and should be interpreted with caution when used as stand-alone subscores.

In the initial 54-item specification, F9 (behavioral impulsivity) exhibited weak psychometric properties (*α* = 0.596), with item B6 showing a low standardized loading (β = 0.263) in contrast to B7 and B8. Two explanations are plausible: the highly regulated cadet environment may impose external constraints on self-regulatory demand and behavioral expression [36], and B6 may be particularly susceptible to social desirability in military self-report [37].

Although the early EFA literature has recommended a minimum of three items per factor [22], two-indicator factors are widely regarded as acceptable within larger multi-factor CFA models that provide the information needed for identification [12,38,39]. F9 is identified through its embedding in D2 and meets all key empirical criteria. Nevertheless, expanding F9 to at least three well-performing indicators remains a priority for the next revision cycle, and users are advised to interpret F9 in conjunction with other D2 factors rather than as a stand-alone subscore.

### 4.4. Convergent, Discriminant, and Criterion Validity

Convergent validity was supported by CFA standardized loadings, CR, and AVE estimates that met conventional thresholds at both the first- and second-order levels [32]. Discriminant validity was evaluated using two complementary criteria. At the first-order level, √AVE values (range 0.789–0.948) exceeded the absolute inter-factor correlations for all 13 factors. At the second-order level, √AVE for D2 (0.904) and D3 (0.918) each exceeded the absolute latent interdimension correlations; √AVE for D1 (0.789) was marginally exceeded by the latent D1 ↔ D2 correlation (0.850), while the absolute D1 ↔ D3 correlation (|−0.780|) fell just below √AVE(D1). As a complementary index, HTMT ratios [33] fell well below the conservative 0.85 threshold for all three dimension pairs (D1–D2 = 0.735; D1–D3 = 0.724; D2–D3 = 0.567), providing converging evidence for empirical distinguishability. The pattern is consistent with the theoretically expected coupling of stress exposure, vulnerability, and protective resources under the diathesis–stress–resource framework [5,7].

Correlations with the PHQ-9, GAD-7, and WHO-5 were in the predicted directions and magnitudes (Section 3.6.3; Table 7), supporting criterion-related validity. D1 and D2 correlated positively with depression and anxiety and negatively with well-being; D3 showed the opposite pattern. The full-scale correlations with the PHQ-9 (*r* = 0.700) and GAD-7 (*r* = 0.685) indicate substantial shared variance without construct redundancy, consistent with the positioning of the MCMHRS as an upstream risk-and-resource instrument. Among first-order factors, F7, F8, and F13 showed the strongest associations with the external criteria, paralleling their prominent role in transdiagnostic models of internalizing psychopathology [34,35].

### 4.5. Comparison with Existing Instruments and Implications for Measurement-Based Care

Compared with generic symptom checklists such as the SCL-90—widely used in Chinese military populations [40,41] but designed to quantify current symptomatology—the MCMHRS shifts the assessment focus to the upstream balance of risk exposure, vulnerability, and resources, supporting preventive rather than retrospective use. Its item content is also anchored in cadet-specific domains (training pressure, hierarchical dynamics, institutional adaptation, professional identity, team support), consistent with evidence from Chinese military norms indicating heterogeneity across personnel categories [41]. Compared with resilience-focused instruments such as the CD-RISC [9], which capture only protective traits, and with stress-focused inventories that omit vulnerability and resources, the MCMHRS integrates all three components in a single hierarchical framework, reducing respondent burden and enhancing cross-construct comparability [10]. The MCMHRS is not intended to replace symptom measures such as the PHQ-9 or GAD-7, but to complement them by providing upstream risk-and-resource information to inform targeted prevention and early intervention.

Within the framework of measurement-based mental healthcare, the MCMHRS offers a brief, multilevel instrument that, once its predictive validity is established through longitudinal studies, may be suitable for routine periodic administration alongside standard physical and psychological evaluations. By providing simultaneous indices of risk exposure, vulnerability, and protective resources, the instrument aims to bridge psychometric rigor with future real-world utility in cadet mental healthcare.

### 4.6. Limitations and Future Directions

Several limitations should be acknowledged when interpreting the present findings. First, the cross-sectional design precludes evaluation of test–retest reliability and of predictive validity for incident depression, anxiety, attrition, or occupational impairment; longitudinal designs are needed to establish the prognostic utility of the instrument.

Second, formal multi-group CFA tests of measurement invariance across gender were not feasible in the present study because the female subsample (*n* = 61 in the validation subsample) fell below the minimum group size typically recommended for models of this complexity [12]. Measurement invariance across academic year, household-registration background, and cadet leadership status was likewise not formally tested and is a priority for subsequent work, ideally with targeted oversampling of female cadets.

Third, two localized psychometric issues warrant caution. F9 (behavioral impulsivity) is indicated by only two items in the final model; although this specification is methodologically acceptable within larger multi-factor structural equation models [12,39] and F9 met all empirical criteria (*α* = 0.788; CR = 0.876; AVE = 0.783), expanding F9 to at least three well-performing indicators is a priority for the next validation cycle. In addition, because the removal of B6 and the estimation of the final 53-item model both drew on the validation subsample, the M2 specification has not been strictly cross-validated in an independent sample; replication in a new cadet cohort is a priority for future work. Relatedly, although HTMT-based discriminant validity was supported (all pairs < 0.85), √AVE for D1 (0.789) was marginally exceeded by the D1 ↔ D2 latent correlation (0.850), indicating that the strict Fornell–Larcker criterion was not fully met for D1. This likely reflects the theoretically expected coupling of environmental stressors with individual vulnerability in the highly regimented cadet environment; targeted refinement of D1 items with strong appraisal components could further sharpen this boundary. Relatedly, although the second-order model was retained on grounds of parsimony, information-criterion evidence, and theory, it fitted significantly worse than the first-order correlated model in the strict exact-fit sense, and AIC and aBIC favored the latter; the adequacy of the second-order representation should therefore be re-examined in independent samples.

Fourth, because all measures were self-reported at a single time point, common method variance cannot be ruled out; future multi-method designs incorporating informant ratings, behavioral tasks, or clinical interview data are needed to confirm the observed associations.

Building on these considerations, priority future work includes (a) longitudinal validation for test–retest reliability and predictive validity; (b) measurement invariance testing across demographic and contextual subgroups, contingent on targeted recruitment; (c) cross-institutional replication in independent cadet samples to consolidate the hierarchical structure; and (d) refinement of F9 through expanded item generation and possible behavioral or peer-report indices. Together, these efforts would consolidate the MCMHRS for broad application in cadet mental health assessment.

## 5. Conclusions

The Military Cadets’ Mental Health Risk Scale (MCMHRS) is a 53-item, theoretically grounded instrument comprising 13 first-order factors nested within three second-order dimensions—environmental stressors, individual vulnerabilities, and protective factors. In two independent subsamples (calibration *n* = 244; validation *n* = 366) drawn from 610 Chinese cadets, the MCMHRS demonstrated a well-fitting hierarchical structure (χ^2^/*df* = 1.69, RMSEA = 0.043, CFI = 0.978), excellent internal consistency (*α* = 0.968), and theoretically coherent convergent, discriminant, and criterion-related validity against the PHQ-9, GAD-7, and WHO-5. By integrating risk exposure, individual susceptibility, and protective resources within a single hierarchical framework anchored in the stress ecology of military academies, the MCMHRS provides an initial foundation for measurement-based assessment in cadet mental healthcare. Its utility for screening, intervention planning, and longitudinal monitoring awaits confirmation in longitudinal validation studies. Future work should establish test–retest reliability and predictive validity, examine measurement invariance across demographic subgroups, replicate findings in independent cadet samples, and refine the behavioral impulsivity factor.

## Figures and Tables

**Table 1:** Demographic characteristics of the total sample (*N* = 610).

Variable	Category	*n*	%
Gender	Male	504	82.6
	Female	106	17.4
Academic year	Year 1	264	43.3
	Year 2	229	37.5
	Year 3	117	19.2
Household registration	Urban	336	55.1
	Rural	274	44.9
Only-child status	Yes	208	34.1
	No	402	65.9
Cadet leadership role	Yes	266	43.6
	No	344	56.4
Age (years)	*M* ± *SD*	19.45 ± 1.42	(Range: 18–25)

**Table 2:** Demographic equivalence between calibration and validation subsamples (*N* = 610).

Variable	Category	Calibration (*n* = 244)	Validation (*n* = 366)	χ^2^/*t*	*df*	*p*
Gender	Male	199 (81.6%)	305 (83.3%)	0.322	1	0.571
	Female	45 (18.4%)	61 (16.7%)			
Academic year	Year 1	102 (41.8%)	162 (44.3%)	1.696	2	0.428
	Year 2	89 (36.5%)	140 (38.2%)			
	Year 3	53 (21.7%)	64 (17.5%)			
Household registration	Urban	142 (58.2%)	194 (53.0%)	1.595	1	0.207
	Rural	102 (41.8%)	172 (47.0%)			
Only-child status	Yes	86 (35.2%)	122 (33.3%)	0.238	1	0.625
	No	158 (64.8%)	244 (66.7%)			
Cadet leadership role	Yes	112 (45.9%)	154 (42.1%)	0.871	1	0.351
	No	132 (54.1%)	212 (57.9%)			
Age (years)	*M* ± *SD*	19.47 ± 1.53	19.44 ± 1.34	0.268	608	0.788

**Table 3:** Item reduction across development stages.

Stage	Procedure	Items Removed	Retained
1. Item generation	Literature review + focus groups	—	78
2. Delphi validation (*n* = 15)	17 removed (low I-CVI/redundancy); 3 added	−14 (net)	64
3. Item analysis (*n* = 244)	C7 removed (corrected item-total *r* = 0.270)	−1	63
4. EFA (*n* = 244)	D1: A4, A16, A23 removed (cross-loading); D2: B9, B10, B17–B20 removed (structure did not replicate)	−9	54
5. CFA sensitivity analysis (*n* = 366)	B6 removed (β = 0.263 in initial model; F9 *α* improved from 0.596 to 0.788 after removal)	−1	53

*Note*. I-CVI = item-level content validity index; EFA = exploratory factor analysis; CFA = confirmatory factor analysis.

**Table 4:** Fit indices and information criteria of competing CFA models (*n* = 366).

**Panel A. Primary estimation (WLSMV, ordered categorical indicators)**										
**Model**	**χ2**	***df***	**χ2/*df***	**RMSEA**	**90% CI**	**CFI**	**TLI**	**SRMR**	**Parameters**	***Δ*χ2 vs. M1 (*df*)**
M0: Single factor	6676.668	1325	5.04	0.105	[0.103, 0.108]	0.871	0.866	0.112	261	—
M1: 13 correlated factors	1957.531	1247	1.57	0.039	[0.036, 0.043]	0.983	0.981	0.044	339	—
M2: Second-order, 3 dimensions	2212.431	1309	1.69	0.043	[0.040, 0.047]	0.978	0.977	0.057	277	236.490 (62) ***
**Panel B. Re-estimation for information criteria (MLR, continuous indicators)**										
**Model**	**χ2 (Scaled)**	***df***	**χ2/*df***	**Scaling Factor**	**Parameters**	**AIC**		**BIC**		**aBIC**
M0: Single factor	5690.776	1325	4.29	1.4075	159	41,336.643		41,957.162		41,452.718
M1: 13 correlated factors	2022.998	1247	1.62	1.3462	237	36,206.560		37,131.484		36,379.577
M2: Second-order, 3 dimensions	2198.450	1309	1.68	1.3507	175	36,328.592		37,011.553		36,456.347

*Note.* CI = confidence interval; RMSEA = root mean square error of approximation; CFI = comparative fit index; TLI = Tucker–Lewis index; SRMR = standardized root mean square residual; AIC = Akaike information criterion; BIC = Bayesian information criterion; aBIC = sample-size-adjusted BIC. All three models were estimated on the same final 53-item indicator set (i.e., after removal of B6, as described in Section 3.4), ensuring strictly like-for-like comparison. Panel A reports the primary solutions, in which items were treated as ordered categorical indicators and estimated with WLSMV; the *Δ*χ2 value was obtained with the Mplus DIFFTEST procedure, as mean- and variance-adjusted χ2 statistics cannot be compared by simple subtraction. Because likelihood-based information criteria are undefined under WLSMV, Panel B reports re-estimations of the identical models with maximum likelihood with robust standard errors (MLR), treating items as continuous; the χ2 values in Panel B are Satorra–Bentler scaled statistics and the parameter counts additionally include 53 item intercepts. *** *p* < 0.001.

**Table 5:** Reliability indices of the MCMHRS (validation subsample, *n* = 366).

Level/Factor	*k*	*α*	Split-Half	CR	AVE
D1 Environmental stressors	23	0.926	0.860	0.906	0.622
F1 Military training pressure	4	0.893	0.882	0.928	0.778
F2 Academic competition pressure	4	0.873	0.835	0.910	0.711
F3 Military cultural adaptation	4	0.869	0.841	0.929	0.770
F4 Interpersonal pressure	3	0.729	0.685	0.893	0.733
F5 Career prospect pressure	4	0.892	0.895	0.929	0.769
F6 Family-of-origin pressure	4	0.743	0.730	0.868	0.622
D2 Individual vulnerabilities	12	0.933	0.901	0.930	0.818
F7 Emotional instability	5	0.892	0.867	0.939	0.760
F8 Negative cognitive pattern	5	0.875	0.864	0.929	0.724
F9 Behavioral impulsivity	2	0.788	0.788	0.876	0.783
D3 Protective factors	18	0.957	0.921	0.952	0.842
F10 Psychological resilience	5	0.931	0.898	0.962	0.839
F11 Social support	4	0.817	0.792	0.901	0.697
F12 Professional identity	4	0.935	0.917	0.972	0.898
F13 Positive coping	5	0.913	0.900	0.949	0.799
Full scale	53	0.968	—	—	—

*Note. k* = number of items; *α* = Cronbach’s alpha; split-half = Spearman–Brown split-half coefficient; CR = composite reliability; AVE = average variance extracted. CR and AVE were not computed at the full-scale level given the hierarchical structure of the instrument. D2 and full-scale statistics reflect the 53-item final version after removal of B6 (see Section 3.4).

**Table 6:** Discriminant validity of second-order dimensions (*n* = 366).

Dimension	√AVE	D1	D2	D3
D1 Environmental stressors	0.789	—	0.850	−0.780
D2 Individual vulnerabilities	0.904	0.735	—	−0.746
D3 Protective factors	0.918	0.724	0.567	—

*Note.* The √AVE column reports the square root of the average variance extracted for each dimension. Values above the diagonal are latent interdimension correlations from CFA; D3 correlations have been sign-adjusted so that higher D3 = greater protective resources, consistent with Table 7 and Section 3.6.3. Values below the diagonal are HTMT ratios [33]; HTMT values < 0.85 support discriminant validity. All correlations were statistically significant at *p* < 0.001.

**Table 7:** Pearson correlations between the MCMHRS and external criterion measures (validation subsample, *n* = 366).

MCMHRS Indicator	PHQ-9	GAD-7	WHO-5
D1 Environmental stressors	0.615 ***	0.605 ***	−0.497 ***
F1 Military training pressure	0.447 ***	0.450 ***	−0.406 ***
F2 Academic competition pressure	0.526 ***	0.539 ***	−0.470 ***
F3 Military cultural adaptation	0.472 ***	0.468 ***	−0.347 ***
F4 Interpersonal pressure	0.366 ***	0.339 ***	−0.265 ***
F5 Career prospect pressure	0.418 ***	0.425 ***	−0.306 ***
F6 Family-of-origin pressure	0.497 ***	0.416 ***	−0.352 ***
D2 Individual vulnerabilities (12 items)	0.672 ***	0.679 ***	−0.524 ***
F7 Emotional instability	0.633 ***	0.664 ***	−0.522 ***
F8 Negative cognitive pattern	0.637 ***	0.615 ***	−0.496 ***
F9 Behavioral impulsivity (2 items)	0.478 ***	0.489 ***	−0.297 ***
D3 Protective factors	−0.583 ***	−0.553 ***	0.514 ***
F10 Psychological resilience	−0.525 ***	−0.517 ***	0.477 ***
F11 Social support	−0.507 ***	−0.462 ***	0.460 ***
F12 Professional identity	−0.394 ***	−0.343 ***	0.323 ***
F13 Positive coping	−0.600 ***	−0.587 ***	0.522 ***
Full scale total (53 items)	0.700 ***	0.685 ***	−0.576 ***

*Note.* PHQ-9 = Patient Health Questionnaire—9; GAD-7 = Generalized Anxiety Disorder—7; WHO-5 = WHO-5 Well-Being Index. D3 is scored in its original direction (higher = greater protective resources). *** *p* < 0.001.

## Data Availability

The data presented in this study are not publicly available because of participant privacy and ethical restrictions arising from the sensitive nature of mental health information collected within a military academy setting. De-identified data, as well as the full Chinese wording and scoring key of the MCMHRS, may be requested from the corresponding author subject to approval by the Medical Ethics Committee of the Army Medical University and completion of a data-use agreement.

## Supplementary Materials

The following supporting information can be downloaded at https://www.mdpi.com/article/10.3390/healthcare14172730/s1, Table S1. Summary of analytic procedures, criteria, and decision thresholds; Table S2. Item analysis results for all 64 initial items (calibration subsample, *n* = 244); Table S3. Standardized first-order factor loadings for the final 53-item M2 model (*n* = 366); Table S4. Second-order factor loadings of the 13 first-order factors on the three second-order dimensions (*n* = 366); Table S5. EFA sampling adequacy and factor-retention overview (calibration subsample, *n* = 244); Table S6. EFA pattern matrix and communalities for D1 environmental stressors (*n* = 244); Table S7. EFA pattern matrix and communalities for D2 individual vulnerabilities (*n* = 244); Table S8. EFA pattern matrix and communalities for D3 protective factors (*n* = 244).

## Abbreviations

The following abbreviations are used in this manuscript:

MCMHRS	Military Cadets’ Mental Health Risk Scale
D1	Environmental Stressors (Second-Order Dimension)
D2	Individual Vulnerabilities (Second-Order Dimension)
D3	Protective Factors (Second-Order Dimension)
F1–F13	First-Order Factors of the MCMHRS
EFA	Exploratory Factor Analysis
CFA	Confirmatory Factor Analysis
KMO	Kaiser–Meyer–Olkin (Measure of Sampling Adequacy)
WLSMV	Weighted Least Squares Mean- and Variance-Adjusted Estimator
χ^2^/df	Normed Chi-Square (Chi-Square Divided by Degrees of Freedom)
RMSEA	Root Mean Square Error of Approximation
CFI	Comparative Fit Index
TLI	Tucker–Lewis Index
SRMR	Standardized Root Mean Square Residual
CR	Composite Reliability
AVE	Average Variance Extracted
HTMT	Heterotrait–Monotrait ratio (of Correlations)
I-CVI	Item-level Content Validity Index
S-CVI/Ave	Scale-level Content Validity Index, Averaging Method
PHQ-9	Patient Health Questionnaire-9
GAD-7	Generalized Anxiety Disorder-7
WHO-5	World Health Organization Five Well-Being Index
SCL-90	Symptom Checklist–90
CD-RISC	Connor–Davidson Resilience Scale
MLR	Maximum Likelihood With Robust Standard Errors
AIC	Akaike Information Criterion
BIC	Bayesian Information Criterion
aBIC	Sample-Size-Adjusted BIC
