# Supplementary material for: Development and Psychometric Evaluation of the Military Cadets’ Mental Health Risk Scale (MCMHRS) in Chinese Military Academy Cadets
Source: Healthcare (Basel). 2026 Aug 27;14(17):2730. doi: 10.3390/healthcare14172730 (PMC13565071; doi:10.3390/healthcare14172730)
Supplement: Supplementary file 1 [file healthcare-14-02730-s001.zip › healthcare-4438927-supplementary.pdf]

healthcare

MDPI

Article

Development and Psychometric Evaluation of the Military Cadets' Mental Health Risk Scale (MCMHRS) in Chinese Military Academy Cadets

Table S1. Summary of Analytic Procedures, Criteria, and Decision Thresholds

Analytic step	Subsample	Procedure / Index	Decision criterion	Reference
Item analysis	Calibration (*n* = 244)	Critical ratio (top vs. bottom 27%)	CR ≥ 3.0, $p < 0.001$	[21]
		Corrected item-total correlation	r ≥ 0.30	[21]
EFA	Calibration (*n* = 244)	Kaiser–Meyer–Olkin measure	KMO ≥ 0.70	[24]
		Bartlett's test of sphericity	$p < 0.001$	[24]
		Extraction: principal axis factoring	—	[22]
		Rotation: Promax (kappa = 4)	—	[22]
		Factor retention	Eigenvalue ≥ 1, scree, parallel analysis, theory	[23]
		Primary factor loading	≥ 0.40	[24]
		Cross-loading difference	≥ 0.20	[24]
		Communality	≥ 0.20	[24]
CFA	Validation (*n* = 366)	Estimator: WLSMV	—	[25]
		χ^2/df	< 3.0	[26]
		RMSEA	≤ 0.06 good; ≤ 0.08 acceptable	[26]
		CFI / TLI	≥ 0.95 good; ≥ 0.90 acceptable	[26]
		SRMR	≤ 0.08	[26]
		Nested model comparison	ΔCFI < 0.01; ΔRMSEA < 0.015; ΔSRMR < 0.030	[27]
		sted comparison of M1 vs. M2: DIFFTEST (WLSMV-adjusted χ^2 difference)	$p < 0.05$ indicates significant deterioration in exact fit; interpreted alongside approximate fit indices	[28]
		Target coefficient (χ^2 first-order / χ^2 higher-order)	Higher values indicate greater covariation explained by the higher-order structure	[29]
		Information criteria (AIC, BIC, aBIC) from MLR re-estimation	Lower values preferred	[20]
Reliability	Validation (*n* = 366)	Cronbach's α	≥ 0.70 acceptable; ≥ 0.80 good; ≥ 0.90 excellent	[21]
		Spearman–Brown split-half	≥ 0.70	—
		Composite reliability (CR)	≥ 0.70	[30]
		Average variance extracted (AVE)	≥ 0.50	[31]
Validity	Validation (*n* = 366)	Convergent: CFA loadings	≥ 0.50 acceptable; ≥ 0.70 preferable	[32]
		Discriminant: Fornell–Larcker	√AVE > inter-factor r	[31]
		Criterion-related: Pearson r with PHQ-9, GAD-7, WHO-5	$p < 0.05$ in theorized direction	[32]
Validity	Validation (*n* = 366)	Discriminant: HTMT ratio	HTMT < 0.85 (conservative); < 0.90 (liberal)	[33]

Sensitivity analysis	Validation (*n* = 366)	Post hoc sensitivity analysis alternative CFA models (53-item variant: item B6 removed; 51-item variant: F9 factor removed); estimated in the same validation subsample as the primary CFA	Comparison of fit indices, factor reliability, and criterion correlations	—

Note. CR = critical ratio (item analysis) or composite reliability (reliability analysis), depending on context. EFA = exploratory factor analysis; CFA = confirmatory factor analysis; WLSMV = weighted least squares mean and variance adjusted; RMSEA = root mean square error of approximation; CFI = comparative fit index; TLI = Tucker–Lewis index; SRMR = standardized root mean square residual; AVE = average variance extracted.

Table S2. Item Analysis Results for All 64 Initial Items (Calibration Subsample, n = 244)

Item	CR (*t*-value)	*p*	Corrected item-total *r*	α if deleted	Decision
D1 Environmental stressors subscale (α = 0.923)					
A1	10.088	< 0.001	0.523	0.920	Retained
A2	9.412	< 0.001	0.498	0.921	Retained
A3	8.756	< 0.001	0.476	0.921	Retained
A4	6.247	< 0.001	0.316	0.924	Retained
A5	11.235	< 0.001	0.572	0.919	Retained
A6	10.674	< 0.001	0.548	0.919	Retained
A7	9.823	< 0.001	0.512	0.920	Retained
A8	8.945	< 0.001	0.487	0.921	Retained
A9	10.412	< 0.001	0.534	0.920	Retained
A10	9.567	< 0.001	0.503	0.920	Retained
A11	11.089	< 0.001	0.561	0.919	Retained
A12	10.234	< 0.001	0.527	0.920	Retained
A13	9.876	< 0.001	0.516	0.920	Retained
A14	8.654	< 0.001	0.471	0.921	Retained
A15	5.002	< 0.001	0.334	0.923	Retained
A16	7.823	< 0.001	0.412	0.922	Retained
A17	10.567	< 0.001	0.541	0.920	Retained
A18	9.234	< 0.001	0.494	0.921	Retained
A19	8.876	< 0.001	0.482	0.921	Retained
A20	10.123	< 0.001	0.521	0.920	Retained
A22	9.456	< 0.001	0.501	0.920	Retained
A23	7.234	< 0.001	0.389	0.922	Retained
A24	10.789	< 0.001	0.553	0.919	Retained
A25	9.876	< 0.001	0.515	0.920	Retained
A26	8.567	< 0.001	0.468	0.921	Retained
A28	10.345	< 0.001	0.532	0.920	Retained
D2 Individual vulnerabilities subscale (α = 0.923)					
B1	11.456	< 0.001	0.587	0.918	Retained
B2	10.823	< 0.001	0.561	0.919	Retained
B3	9.987	< 0.001	0.524	0.920	Retained
B4	11.234	< 0.001	0.578	0.919	Retained
B5	10.456	< 0.001	0.542	0.920	Retained
B6	9.371	< 0.001	0.320	0.922	Retained
B7	10.234	< 0.001	0.531	0.920	Retained
B8	11.089	< 0.001	0.568	0.919	Retained
B9	9.654	< 0.001	0.508	0.920	Retained
B10	10.789	< 0.001	0.551	0.919	Retained

B11	9.456	< 0.001	0.497	0.921	Retained
B12	10.567	< 0.001	0.544	0.920	Retained
B13	11.123	< 0.001	0.573	0.919	Retained
B14	9.876	< 0.001	0.516	0.920	Retained
B15	10.345	< 0.001	0.535	0.920	Retained
B17	8.987	< 0.001	0.479	0.921	Retained
B18	10.678	< 0.001	0.547	0.919	Retained
B19	9.567	< 0.001	0.504	0.920	Retained
B20	10.234	< 0.001	0.528	0.920	Retained
D3 Protective factors subscale (α = 0.943)					
C1	12.456	< 0.001	0.625	0.940	Retained
C2	11.789	< 0.001	0.598	0.941	Retained
C3	12.123	< 0.001	0.612	0.940	Retained
C4	11.567	< 0.001	0.589	0.941	Retained
C6	12.234	< 0.001	0.617	0.940	Retained
C7	7.834	< 0.001	0.270	0.949	Removed[a]
C8	11.987	< 0.001	0.605	0.940	Retained
C9	12.345	< 0.001	0.621	0.940	Retained
C10	11.456	< 0.001	0.584	0.941	Retained
C11	12.567	< 0.001	0.631	0.940	Retained
C12	11.234	< 0.001	0.575	0.941	Retained
C13	12.089	< 0.001	0.609	0.940	Retained
C14	11.678	< 0.001	0.593	0.941	Retained
C15	12.412	< 0.001	0.624	0.940	Retained
C16	11.345	< 0.001	0.579	0.941	Retained
C17	12.234	< 0.001	0.616	0.940	Retained
C18	11.789	< 0.001	0.601	0.940	Retained
C20	12.156	< 0.001	0.613	0.940	Retained
C21	11.523	< 0.001	0.587	0.941	Retained

Note. CR = critical ratio (independent-samples *t*-test comparing top 27% vs. bottom 27% of respondents on the dimension total score). Corrected item-total *r* = Pearson correlation between the item and the sum of the remaining items in the subscale. Subscale Cronbach's α values reported in subheadings are for the calibration subsample (*n* = 244); slightly different α values in the final scale (Table 5 of the main text) reflect the use of the validation subsample (*n* = 366) and the removal of items during EFA refinement.

[a] Item C7 ("Having a clear life plan and direction") was removed during item analysis due to a corrected item-total correlation of 0.270 (below the 0.30 threshold) and an increase in Cronbach's α from 0.943 to 0.949 upon its deletion.

Table S3. Standardized First-Order Factor Loadings for the Final 53-item M2 Model (*n* = 366)

Factor	Item	Standardized loading (β)	*SE*	*t*	*p*	R^2
F1 Military training pressure	A1	0.837	0.022	38.163	< 0.001	0.700
	A2	0.948	0.013	70.401	< 0.001	0.900
	A3	0.891	0.018	48.821	< 0.001	0.794
	A28	0.848	0.033	25.542	< 0.001	0.719
F2 Academic competition pressure	A5	0.876	0.025	34.501	< 0.001	0.767
	A6	0.787	0.029	27.392	< 0.001	0.620
	A7	0.880	0.024	36.715	< 0.001	0.774
	A8	0.825	0.028	29.663	< 0.001	0.681
F3 Military cultural adaptation	A9	0.910	0.027	33.886	< 0.001	0.829
	A10	0.827	0.029	28.445	< 0.001	0.684
	A11	0.930	0.017	54.322	< 0.001	0.864
	A12	0.837	0.025	33.443	< 0.001	0.702

F4 Interpersonal pressure	A13	0.869	0.034	25.441	< 0.001	0.755
	A14	0.895	0.031	28.577	< 0.001	0.801
	A15	0.803	0.042	19.219	< 0.001	0.644
F5 Career prospect pressure	A17	0.824	0.025	32.418	< 0.001	0.679
	A18	0.849	0.021	40.068	< 0.001	0.721
	A19	0.911	0.020	46.031	< 0.001	0.830
	A20	0.919	0.019	49.306	< 0.001	0.845
F6 Family-of-origin pressure	A22	0.740	0.044	16.862	< 0.001	0.548
	A24	0.793	0.038	21.018	< 0.001	0.629
	A25	0.836	0.041	20.519	< 0.001	0.699
	A26	0.782	0.039	19.977	< 0.001	0.612
F7 Emotional instability	B1	0.809	0.031	26.217	< 0.001	0.655
	B2	0.910	0.017	52.173	< 0.001	0.828
	B3	0.882	0.020	45.061	< 0.001	0.778
	B4	0.835	0.026	32.701	< 0.001	0.698
	B5	0.918	0.018	51.364	< 0.001	0.842
F8 Negative cognitive pattern	B11	0.809	0.028	28.990	< 0.001	0.656
	B12	0.858	0.022	39.619	< 0.001	0.736
	B13	0.906	0.018	49.155	< 0.001	0.821
	B14	0.894	0.022	40.161	< 0.001	0.799
	B15	0.779	0.032	24.512	< 0.001	0.606
F9 Behavioral impulsivity	B7	0.788	0.029	27.134	< 0.001	0.621
	B8	0.971	0.026	37.112	< 0.001	0.943
F10 Psychological resilience	C1	0.890	0.017	51.607	< 0.001	0.792
	C2	0.939	0.013	73.159	< 0.001	0.881
	C3	0.943	0.015	63.713	< 0.001	0.889
	C4	0.940	0.012	78.448	< 0.001	0.883
	C6	0.865	0.017	52.356	< 0.001	0.749
F11 Social support	C8	0.787	0.029	27.377	< 0.001	0.620
	C9	0.810	0.026	31.588	< 0.001	0.657
	C10	0.794	0.027	29.478	< 0.001	0.631
	C11	0.939	0.016	58.968	< 0.001	0.882
F12 Professional identity	C12	0.929	0.012	76.798	< 0.001	0.863
	C13	0.970	0.006	150.286	< 0.001	0.941
	C14	0.917	0.014	67.256	< 0.001	0.841
	C15	0.973	0.011	87.608	< 0.001	0.946
F13 Positive coping	C16	0.869	0.019	46.129	< 0.001	0.754
	C17	0.933	0.012	75.343	< 0.001	0.871
	C18	0.877	0.017	50.822	< 0.001	0.769
	C20	0.914	0.013	71.830	< 0.001	0.835
	C21	0.876	0.018	48.855	< 0.001	0.768

Note. Item B6 (initial $\beta = 0.263$) was removed based on the sensitivity analysis reported in main text Section 3.4; loadings for the remaining F9 indicators (B7, B8) shown here reflect the final M2 specification.

Table S4. Second-Order Factor Loadings of the 13 First-Order Factors on the Three Second-Order Dimensions ($n = 366$)

Second-order dimension	First-order factor	Standardized loading (β)	*SE*	*t*	*p*	R^2
D1 Environmental stressors	F1 Military training pressure	0.730	0.030	24.040	< 0.001	0.532
	F2 Academic competition pressure	0.810	0.024	34.418	< 0.001	0.655

	F3 Military cultural adaptation	0.785	0.027	29.018	< 0.001	0.617
	F4 Interpersonal pressure	0.773	0.036	21.492	< 0.001	0.597
	F5 Career prospect pressure	0.813	0.023	34.910	< 0.001	0.661
	F6 Family-of-origin pressure	0.817	0.034	23.734	< 0.001	0.668
D2 Individual vulnerabilities	F7 Emotional instability	0.936	0.015	62.936	< 0.001	0.876
	F8 Negative cognitive pattern	0.958	0.013	72.249	< 0.001	0.918
	F9 Behavioral impulsivity	0.812	0.033	24.085	< 0.001	0.659
D3 Protective factors	F10 Psychological resilience	0.905	0.014	65.248	< 0.001	0.819
	F11 Social support	0.923	0.011	81.924	< 0.001	0.851
	F12 Professional identity	0.866	0.020	44.186	< 0.001	0.749
	F13 Positive coping	0.973	0.008	114.964	< 0.001	0.947

Note. Estimator: weighted least squares mean and variance adjusted (WLSMV). All loadings are standardized using the STDYX standardization in Mplus 8.3. *SE* = standard error; *t* = *z*-equivalent test statistic (estimate / *SE*); R^2 = squared standardized loading, representing the proportion of variance in the indicator accounted for by its factor.

Table S5. EFA Sampling Adequacy and Factor-Retention Overview (Calibration Subsample, *n* = 244)

Dimension	Items entered	KMO	Bartlett's χ^2 (df), p	Factors extracted	Cumulative variance	Retention evidence
D1 Environmental stressors	26	0.894	4087.05 (325), < 0.001	6	67.26%	Eigenvalues ⩾ 1.0, scree plot, and parallel analysis all supported a six-factor solution.
D2 Individual vulnerabilities	19	0.921	2480.24 (171), < 0.001	3	50.91%	Scree plot and parallel analysis supported a three-factor solution; the a priori "maladaptive coping" factor did not emerge as a distinct dimension (see Section 3.3 of the main text).
D3 Protective factors	18	0.928	3770.04 (153), < 0.001	4	70.53%	Eigenvalues ⩾ 1.0, scree plot, and parallel analysis all supported a four-factor solution.

Note. All values are from the calibration subsample. Extraction: principal axis factoring; rotation: Promax (kappa = 4). Item-level pattern matrices and communalities are reported in Tables S6 (D1), S7 (D2), and S8 (D3).

Table S6. EFA Pattern Matrix and Communalities for D1 Environmental Stressors (*n* = 244)

Factor	Item	Loading	h^2
F1 Military training pressure	A1	0.706	0.693
	A2	0.769	0.737
	A3	0.900	0.788
	A28	0.793	0.548
F2 Academic competition pressure	A5	0.919	0.717
	A6	0.796	0.727
	A7	0.693	0.596
	A8	0.628	0.552
F3 Military cultural adaptation	A9	0.726	0.693
	A10	0.977	0.828
	A11	0.825	0.721
	A12	0.510	0.621
F4 Interpersonal pressure	A13	0.793	0.780
	A14	0.845	0.766

	A15	0.630	0.367
F5 Career prospect pressure	A17	0.807	0.694
	A18	0.902	0.723
	A19	0.832	0.782
	A20	0.888	0.823
F6 Family-of-origin pressure	A22	0.722	0.553
	A24	0.750	0.578
	A25	0.788	0.602
	A26	0.753	0.579

Note. Principal axis factoring with Promax rotation (kappa = 4); sampling adequacy and factor-retention details are reported in Table S5. Items A4, A16, and A23 were removed for cross-loading violations and are not shown. Loadings are primary loadings from the pattern matrix; h² = communality.

Table S7. EFA Pattern Matrix and Communalities for D2 Individual Vulnerabilities (*n* = 244)

Factor	Item	Loading	h²
F7 Emotional instability	B1	0.777	0.510
	B2	0.732	0.563
	B3	0.899	0.785
	B4	0.633	0.514
	B5	0.647	0.646
F8 Negative cognitive pattern	B11	0.775	0.623
	B12	0.886	0.725
	B13	0.823	0.679
	B14	0.464	0.483
	B15	0.523	0.352
F9 Behavioral impulsivity	B6	0.578	0.225
	B7	0.555	0.446
	B8	0.632	0.548

Note. Principal axis factoring with Promax rotation (kappa = 4); sampling adequacy and factor-retention details are reported in Table S5. Items B9, B10, B17, B18, B19, and B20 were removed during EFA refinement and are not shown. Item B6 was retained at the EFA stage but subsequently removed after the CFA-based sensitivity analysis (see Section 3.4). Loadings are primary loadings from the pattern matrix; h² = communality.

Table S8. EFA Pattern Matrix and Communalities for D3 Protective Factors (*n* = 244)

Factor	Item	Loading	h²
F10 Psychological resilience	C1	0.606	0.527
	C2	0.957	0.838
	C3	0.978	0.885
	C4	0.692	0.737
	C6	0.755	0.709
F11 Social support	C8	0.527	0.484
	C9	0.582	0.490
	C10	0.845	0.625
	C11	0.787	0.806
F12 Professional identity	C12	0.914	0.826
	C13	0.981	0.899
	C14	0.751	0.689
	C15	0.758	0.732
F13 Positive coping	C16	0.688	0.633

C17	0.806	0.817
C18	0.811	0.659
C20	0.816	0.732
C21	0.718	0.607

Note. Principal axis factoring with Promax rotation (kappa = 4); sampling adequacy and factor-retention details are reported in Table S5. Item C7 was removed during item analysis (see Table S2). Consistent with Section 2.4.1 of the main text, D3 items are shown in their original scoring direction (higher = greater protective resources); D3 items are reverse-coded only when computing the composite total score. Loadings are primary loadings from the pattern matrix; h^2 = communality.

Access to the 53 final items. In line with institutional review procedures governing the use of instruments developed within military academies, the full Chinese wording and scoring key of the 53 MCMHRS items are not distributed in open form. Qualified researchers may request access from the corresponding author, subject to (a) approval by the Medical Ethics Committee of the Army Medical University for the intended research use, and (b) a signed data-use agreement.
